# Multiplexed single cell protein expression analysis in solid tumours using a miniaturised microfluidic assay

**DOI:** 10.1088/2057-1739/aa6aae

**Published:** 2017-04-28

**Authors:** Alastair J Magness, James A Squires, Beatrice Griffiths, Khurum Khan, Amanda Swain, Keith R Willison, David Cunningham, Marco Gerlinger, David R Klug

**Affiliations:** 1 Institute of Chemical Biology, Imperial College London, London SW7 2AZ, United Kingdom; 2 Centre for Evolution and Cancer, The Institute for Cancer Research, 237 Fulham Road, London SW3 6JB, United Kingdom; 3 The Royal Marsden Hospital NHS Foundation Trust, Fulham Road, London SW3 6JJ, United Kingdom; 4 Tumour Profiling Unit, The Institute of Cancer Research, 237 Fulham Road, London SW3 6JB, United Kingdom; 5 These authors contributed equally to the publication.; 6 Author to whom any correspondence should be addressed.

**Keywords:** tumor heterogeneity, xenograft, colorectal cancer, single cell analysis, protein quantification, microfluidics, translational proteomics

## Abstract

Using patient-derived colorectal cancer xenografts, we demonstrate a practicable workflow for single cell proteomics in clinically relevant samples and thus a potential translational route for single cell proteomics into medical diagnostics. Using a microfluidic antibody capture (MAC) chip we measured the expression of the tumour suppressor protein p53 and of its post-translationally modified form phosphorylated at serine-15. Aberrant expression of these has commonly been found in colorectal cancers and has been widely investigated for prognostic significance. Our results show that the MAC technology is viable for quantitatively assessing protein expression and phosphorylation at the single cell level in microscopic amounts of clinically relevant tumour material. Thus, this could become a useful tool in therapeutic-associated single cell protein analysis. We also found dramatic variability of p53 and phosphorylated p53 quantities between individual cancer cells from the same sample, demonstrating the power of this single cell technology to study functional intratumour heterogeneity.

## 1. Introduction

Genetic intratumour heterogeneity has been identified in many cancer types and presents a major diagnostic challenge [1]. Heterogeneity can be present at the macroscale, between large tumour subclones that have significantly expanded and also at the microscale level, between individual cancer cells [2]. Genetic heterogeneity can lead to phenotypic heterogeneity, for example of gene and protein expression levels or protein function [3]. In addition, phenotypic heterogeneity may also occur independently of underlying genetic variation, for example through microenvironmental influences or as a consequence of regulatory processes within cancer cells [4]. Overall, the current lack of insight into the extent of and the mechanisms that establish intratumour heterogeneity and of its clinical relevance is a central hurdle for the development of more effective therapies and biomarkers in oncology. Thus, technologies to assess such phenotypes at the microscale level of single cancer cells are urgently needed. Single cell gene expression profiling has made major progress [5] but technologies to evaluate protein expression and protein modification at the single cell level have been missing. This is important as protein expression levels do not necessarily correlate with mRNA copy number [6], and mRNA analysis gives no information about the post-translational modifications present, which largely determine a protein’s functionality [7–9]. Thus, it is not only copy number, but the form of the copy number that can be important and this is information which mRNA readout cannot provide. Additionally, the dynamics of cellular processes such as signalling networks and interregulatory systems may be poorly represented by proxy measurements of the transcriptome [10], for example, and it may provide very little information about the abundance of proteins whose expression is not or not only regulated at the transcriptional level but also at the level of protein degradation. Importantly, single cell technologies should ideally be able to work with very small quantities of tumour material, both in order to explore the heterogeneity at various levels of spatial resolution, and to make maximum use of often precious and small clinical tissue samples.

The heterogeneity of the expression level of an oncogenic protein, as well as its post-translationally modified isoforms, may have implications for the progression of tumour development, affecting tumour promoting factors such as angiogenesis, the epithelial to mesenchymal transition, resistance to chemotherapy and likelihood of metastasis [11]. Furthermore, identifying heterogeneous expression of protein drug targets may be a useful method of stratifying patients by the likelihood of response to particular therapeutic interventions [12, 13]. The significance of heterogeneous sub-populations can be realised by correlating the proteomic data to therapeutic and other clinically relevant outcomes. Once the significance has been determined, single cell proteomic data could be used to guide treatment, maximising the targeted use of new and emerging therapies such as biologics, or providing information as to the likelihood of resistance or ineffectiveness through effective pharmacoproteomics. In addition, ongoing use of single cell proteomic data could be used to follow disease progression, determine phenotypic changes in the tumour and monitor remission, and could therefore have a significant role across the whole of the therapy, from diagnosis to long-term monitoring. As such the following work represents a step towards an era in which single cell interrogation of protein expression levels is integrated into the wider landscape of molecular characterisation and patient stratification commonly referred to as personalised medicine.

We have previously developed single cell protein analysis methods and have tested and validated them on a range of cell lines [14–16]. These methods are based on microfluidics, with optical trap cell handling and incorporating single molecule detection. Key advantages of this approach are that it is quantitative and robust [17], which may provide a window into protein expression and modification heterogeneity both within and between cell types [18].

Here, we provide feasibility data and describe the workflow for the single cell proteomic and phosphoproteomic analysis of tumour samples with a size similar to that of typical cancer core biopsies. Processing involves tumour disaggregation into a single cell solution, cell sorting with optical tweezers to select only live cells of a chosen type then transferring the cells to individual analysis chamber and analysing them with sufficient accuracy, precision and sensitivity to be able to quantify the levels of selected protein species.

## 2. Methods

### 2.1. The MAC chip

The MAC chip is in essence a miniaturised ELISA assay contained within a nanolitre-scale microfluidic environment allowing protein quantification with single molecule resolution. We have already demonstrated the MAC chip as an effective platform for the absolute and relative quantification of protein copy number in single cells derived from cell lines, but the MAC chip has several features which make it an intrinsically useful platform for the processing of clinical material. These include: (1) a low requirement for primary cells in terms of their absolute number meaning analyses can be performed on scarce or precious samples, or that very small biopsies could provide sufficient material for analysis, opening the possibility for less invasive methods; (2) no fixation is required to perform the assay meaning cells are viable till the point of analysis and thus functional and dynamic studies may be performed; and (3) high selectivity for both viability and specific surface antigen or morphologically defined subpopulations of interest e.g. epithelial or mesenchymal cells, which may be of particular importance when working with processed primary samples that can contain a large proportion of dead material or that may be highly heterogeneous with respect to their cellular constitution. In this paper we demonstrate effective analysis on samples with live cell fractions as low as 1%.

**Figure 1.**
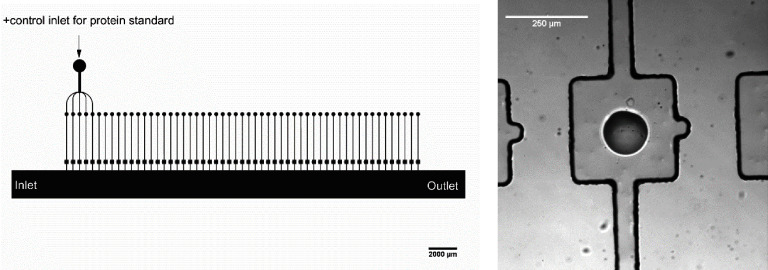
Schematic diagram of the Standard Candle MAC chip variant used in these experiments. The new design features the addition of a set of internal control lanes into which a standard solution positive for the analyte of interest can be flowed, for example recombinant protein at a known concentration, or cell lysate. (Right) Printed antibody spot inside a 300  ×  300 *µ*m MAC chip analysis chamber.

The design of the microfluidic itself consists of a large reservoir channel into which the single cell suspension is loaded, perpendicular to which are 55 channels leading to individual analysis chambers. Cells are moved from the main channel into the analysis chambers via an optical trap. This optical trap allows specific cells to be chosen for analysis based on criteria such as cell surface markers expression and viability as we demonstrate in this paper. The internal volume of the main reservoir channel is approximately 3.3 *µ*l and hence this is the approximate lowest volume that may be processed by the chip and still efficiently filled using the optical trap. A useful modification of the device, the Standard Candle MAC chip (figure 1), contains an additional inlet connected directly to five of these antibody capture chambers and allows the introduction of a positive control for the analyte of interest in order to rule out assay malfunction as the reason for null results when dealing with samples where the expected value of the analyte is unknown. CAD files for the MAC chip design are available in the supplementary information.

**Figure 2.**
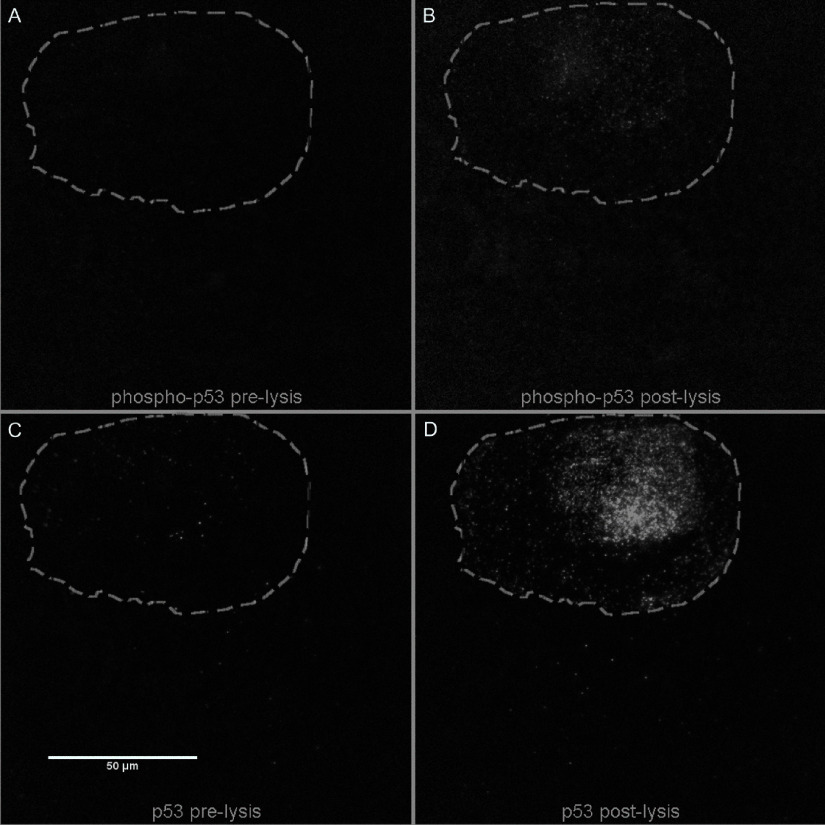
Example multiplex TIRF single molecule data for a single p53 antibody spot viewed under 488 illumination (panels (A) and (B)) and 647 nm illumination (panels (C) and (D)) from a MAC chip experiment. There is a time delay of a few minutes between 488 and 647 channel acquisitions, though with the assay at chemical equilibrium the relative extent of binding between the phospho-p53 and p53 channels is unaffected. The white dotted line approximates the perimeter of the printed antibody spot.

All cellular manipulation and analysis in the MAC chip platform is performed optically and based around a standard inverted microscope. Cells are loaded into analysis chambers from the main sample chamber using an optical trap. Cellular lysis is achieved with a high energy laser pulse into the medium surrounding the cell to produce a cavitation bubble capable of rupturing the cell membrane and releasing its contents into the analysis volume. The assay readout is by total internal reflection (TIRF)-based optical single molecule imaging of the fluorescence at the antibody capture spot (figure 2). Two hours post-lysis, which is sufficient time for the antibody sandwich assay to reach equilibrium [15], the bound protein is imaged using the readout laser and an Andor iXon DU-897E EMCCD camera (Andor Technologies, Ireland).

### 2.2. Xenograft processing

Xenografts were established in the Tumour Profiling Unit at the Institute of Cancer Research from biopsies acquired through the ProspectC and ProspectR colorectal cancer trials at the Royal Marsden Hospital (London, UK). Each trial had been approved by an ethics committee and all patients provided written informed consent before trial participation and biopsy. All tumours were genotyped for TP53 mutations. Mutations are defined according to the main Ensembl transcript for TP53, ENST00000269305.4. A small piece from each biopsy was grafted heterotopically, subcutaneously or under the kidney capsule of CD1 nude female mice. Xenografts included in these experiments were further passaged in the flanks of mice between 1 and 4 times before use. The patient-derived colorectal cancer xenografts (PDXs) were then harvested and dissociated into single cell suspensions in a gentleMACS Octo dissociator using the human Tumour Dissociation Kit (both Miltenyi Biotec) according to the manufacturer’s instructions. Mouse cells were depleted from the cell suspension with the Miltenyi Mouse Cell Depletion kit which contains a mixture of antibodies recognising mouse cells, to remove mouse material from a homogeneous sample via immunomagnetic labelling. An optional red blood cell removal step was omitted, and did not lead to any downstream difficulties during cell selection with the optical trap, however this could be implemented to further clean up samples if desired. Cell viability was checked after the mouse cell depletion with trypan blue staining on a Countess automated cell counter (ThermoFisher Scientific). The viability of the cancer cells in the single cell solution varied over a wide range from 1% to 78%. Dissociated cells were routinely resuspended at a concentration of 2  ×  10^6^ total cells per ml in DMEM/F12 media  +10% FBS for transport to the analysis laboratory, and kept on ice until being introduced into the MAC chip.

In order to distinguish viable from non-viable cells during MAC chip loading, the sample was incubated with 0.04% trypan blue (ThermoFisher Scientific, supplementary figure 1 (stacks.iop.org/CSPO/3/024003/mmedia)) in media, or spun down and resuspended in PBS with LIVE/DEAD^®^ Fixable Violet Dead Cell Stain Kit (ThermoFisher Scientific), on ice for 30 min prior to loading of the sample into the MAC chip. Incubation in PBS must take place for the LIVE/DEAD stain as the presence of extraneous proteins inhibits the efficiency of the dye coupling reaction. After incubation in PBS, cells were resuspended in 4% BSA in PBS for introduction to the MAC chip. Initial experiments were performed with trypan blue, however it has previously been shown that this may increase cellular p53 levels in epithelial cells [19] and we subsequently changed to the LIVE/DEAD stain. The final two PDX derived cell suspensions were also incubated with Alexa-555 labelled anti-EpCAM antibody (clone VU1D9, New England Biolabs UK) to test the feasibility of cell surface marker specific cell selection within the MAC-chip. The specific wavelength of the labelling dyes selected depends on the exact set up of the operator’s system; here we chose dyes compatible with the excitation and emission bands of the LF405/488/532/635-A-000 filter (Semrock) and which did not lead to crosstalk with the fluorescent 488 and 647 detection fluorophores.

On average around 40 *µ*g of solid tumour was processed per microfluidic chip, an estimated value arrived at by multiplying the processed volume of cell suspension (approx. 10 *µ*l), the cell seeding density and an approximate cell mass of 2.29 ng [20].

### 2.3. Microarray printing and microfluidic manufacture

Microfluidic devices were manufactured according to standard Su-8-based photolithography techniques [21, 22]. Briefly, the permanent epoxy negative photoresist Su8-2035 (MicroChem Corp) was spun on a 100 mm Si wafer so as to form a 50 *µ*m-thick layer, which was then soft baked at 65 °C for 1 min, 95 °C for 6.5 min, then exposed to UV light through a high resolution film photomask (Micro Lithography Services Ltd, UK) according to Microchem guidelines, then post-exposure baked at 65 °C for 1 min, 95 °C for 6 min. Uncrosslinked Su-8-2035 was removed with propylene glycol monomethyl ether acetate and the final mould was rinsed with isopropanol prior to vapour deposition of (tridecafluoro-1, 1, 2, 2-tetrahydrooctyl)trichlorosilane (Sigma-Aldrich, UK) in order to passivate the mould and allow easy removal of PDMS copies at later stages. PDMS was mixed 10:1 with curing agent (Sylgard 184 Silicon Elastomer Kit, Dow Corning) then poured on the Su-8 mould and cured at room temperature for 24–48 h until solid.

Prior to assembly of the microfluidic chip the PDMS was punched with fluidic inlets and washed overnight in detergent solution (1% Alconox, Alconox Inc.) then rinsed copiously in isopropanol and dried with nitrogen. Anti-p53 primary antibody (p53-MDM2 ELISA kit, Enzo Life Sciences) was mixed 1:1 with 6XSSC 3 M betaine protein printing buffer and printed on Nexterion Type H coverslips (Schott, UK) using an OmniGrid Micro microarrayer. Antibody immobilisation is via nonspecific physical adsorption. The printed slide and PDMS channels were then assembled using a custom-built alignment rig.

### 2.4. On-chip cell selection and sorting

A disaggregated tumour sample can be highly heterogeneous in terms of the output material, and may contain erythrocytes, dead cells and other debris that are unwanted in the final single cell analysis (supplementary figure 2). Immunofluorescence labelling of cell surface markers in conjunction with viability staining is used to identify specific cellular phenotypes. In these experiments target live, EpCAM-expressing cells are selected from their fluorescence profiles with a manually operated optical trap which is capable of picking up and moving target cells through the microfluidic channels of the device and into the analysis chamber. An expanded panel of antibody marker stains would open the possibility of more complex phenotypic discrimination.

The cell loading process is guided manually by the operator and can be completed within ~30–45 min. The total time from cell loading to completion of cell lysis is approximately 1–1.5 h. Factoring in the time for the tumour dissociation and mouse cell depletion steps (1.5 h) and the subsequent fluorescent labelling step, the total time from the harvest of the xenograft to single cell protein analysis could be as low as 3 h. When the technique is applied to biopsies directly from cancer patients, the analysis time could be reduced to under 2.5 h, as no mouse cell depletion step will be required. It may be possible to reduce this even further and de-skill the assay by automating the sorting process of viable and labelled cells through straightforward algorithms.

### 2.5. Image analysis

Image analysis is performed via custom routines in FIJI [23, 24] which utilise the GDSC Single Molecule Localisation Microscopy plugins from the University of Sussex [25]. Fluorescent single molecules are observed on the antibody capture spot as diffraction-limited intensity peaks. In images where all of these peaks are individually resolvable (e.g. figures 2(A)–(C)), a peak-fitting routine is performed which checks the convergence of local intensity maxima to an ideal 2D Gaussian function approximating the point-spread-function of the microscope optics. Image regions where convergence is attained are designated single molecules while those where it is not are ignored. The total number of single molecules in a given image is then the sum of all intensity maxima where Gaussian peak fitting is successfully performed. For images with a large number of bound molecules (e.g. figure 2(D)), where a substantial proportion of peaks are unresolvable due to the close proximity of other molecules, peak-fitting is unsuitable for counting and instead the total intensity of the image must be divided by the mean intensity of a single secondary molecule. p53 expression is instead given as a background-corrected value corresponding to the increase in single molecule binding at the antibody spot after lysis of the cells.

### 2.6. Target analytes

p53 and pS15-p53 levels are relevant in a variety of human cancers, including colorectal cancers [26], and these were chosen for investigation on the first MAC chip system we have developed that is capable of multiplexed readout. Mutations of TP53 are commonly observed in colorectal cancer, with five ‘hot-spot’ mutations at codons 175, 245, 248, 273 and 282 accounting for over 40% of cases [27].

p53 function is modulated significantly through post-translational modification and S15 phosphorylation is considered to be a key modification in the activation of p53, which acts through a variety of modes to activate and stabilise p53, as well as masking the nuclear export signal on p53, preventing its export out of the nucleus [28].

## 3. Results

Dissociated cancer cells from nine colorectal PDXs were subjected to single cell protein analysis: five from freshly culled mice, while the remainder were taken from frozen stocks of disaggregated cell suspension obtained during previous rounds of harvesting and thawed immediately prior to the MAC chip experiment. All tumours had been genotyped for TP53 mutations before samples were used for any experiments.

Multiple samples from the same PDX lines were examined in three cases. PDX-98-P4 and PDX130-P2 were repeated using thawed material of the originally tested xenograft. These samples are indicated with the addition of an asterisk as PDX-98-P4^*^ and PDX130-P2^*^. A single PDX, PDX97, was repeated using fresh material from a different mouse to the one originally tested, though the source of the xenograft was the same patient and the xenograft had undergone the same number of passages through mouse hosts. We denote these PDX97-P2 and PDX97-P2^**^ respectively. These repeat experiments demonstrate the robustness of the methodology in analysing different sources of material. A summary of the PDX information is provided in table 1. Mean human cell viability in the dissociated samples was 38% (supplementary figure 3).

**Table 1.** Summary of PDXs used in experiments. A single asterix^*^ indicates A PDX experiment using frozen material; a double asterix^**^ indicates a fresh repeat of a xenograft with the same PDX identifier.

Sample	p53 mutation status	Xenograft source	Assay	Pre-selection human cell viability (%)	Selection
PDX97-P2	—	Fresh	p53	10	None
PDX98-P4	—	Fresh	p53	39	Trypan blue
PDX130-P2	TP53 R175H	Fresh	p53	63	Trypan blue
PDX156-P1	TP53 R342 truncation	Fresh	p53	62	Trypan blue
PDX73-P2^*^	TP53 R175H	Frozen	p53 + phospho-p53	24	Trypan blue
PDX100-P2^*^	—	Frozen	p53	78	Live/Dead
PDX97-P2^**^	—	Fresh	p53 + phospho-53	54	Live/Dead
PDX98-P4^*^	—	Frozen	p53 + phospho-53	10	Live/Dead, EpCAM
PDX130-P2^*^	TP53 R175H	Frozen	p53 + phospho-53	1	Live/Dead, EpCAM

Single cells from all nine samples were analysed for p53 protein expression and of these a subset of four were also subjected to the additional phosphorylated p53 (S15) measurement. Two 55-chamber Standard Candle MAC chips were used per sample, into which cells were loaded and lysed sequentially. The only exception was PDX130-P2, which was loaded into three Standard Candle MAC chips.

### 3.1. p53 measurements

**Figure 3.**
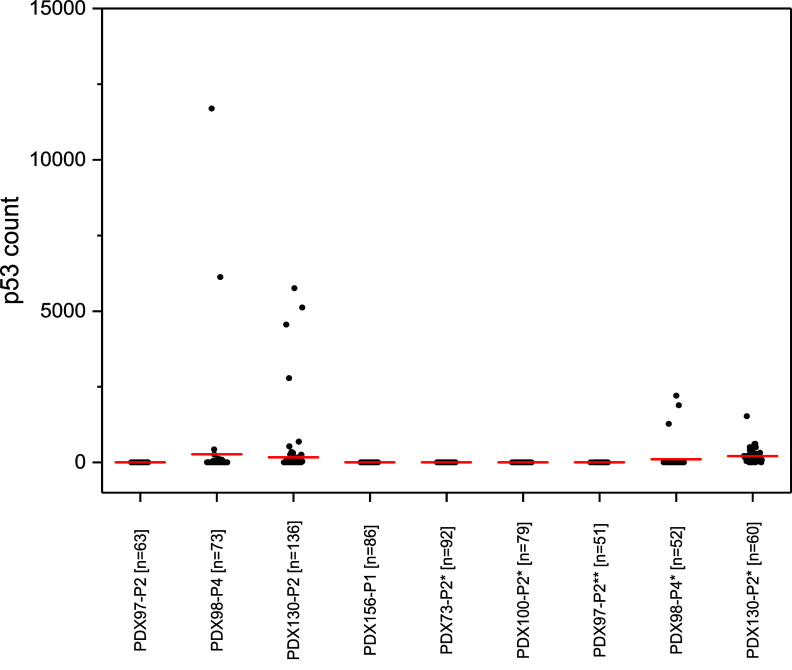
Heterogeneous single cell p53 signal in human colorectal cancer mouse xenograft cells. *n*  =  total number of cells analysed in the experiment.

**Figure 4.**
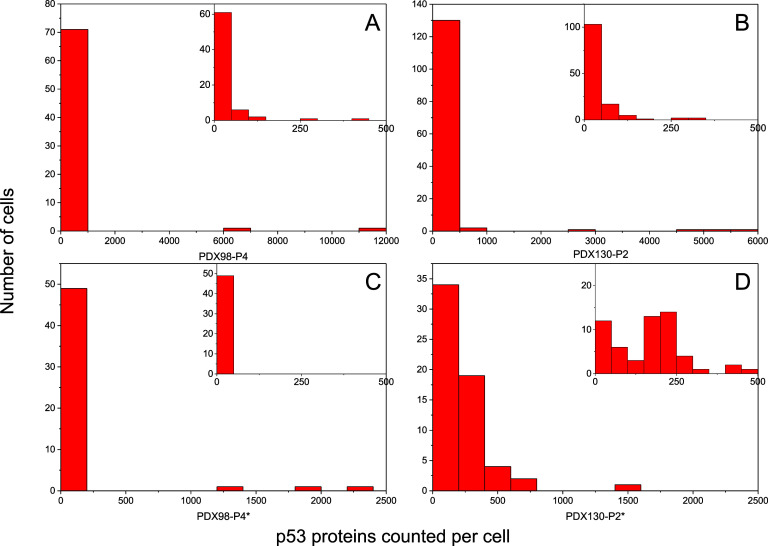
p53 expression distribution in four PDX samples that contained cells with high p53 expression levels. Note the different scales of the *x* and *y*-axes. (Inset graphs) Zoom of low expressing region, below 500 counted proteins.

**Figure 5.**
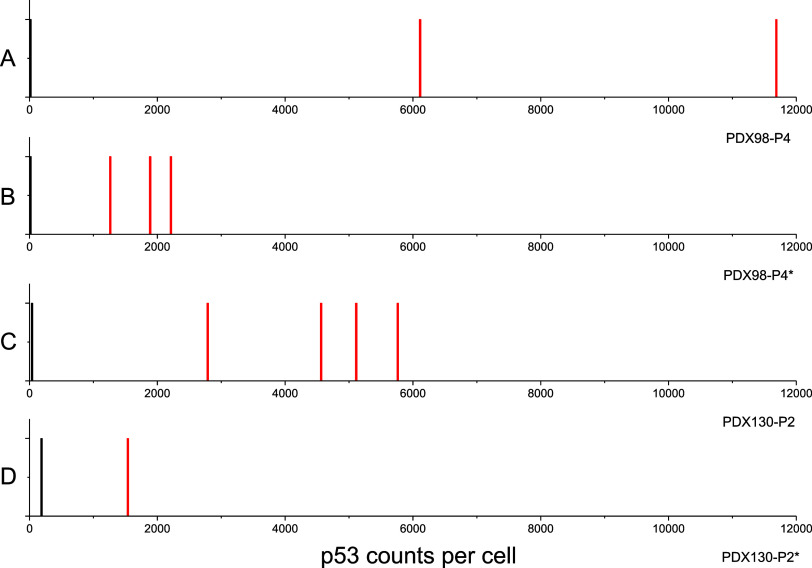
Outlier cells (red vertical bars) express many multiples of the mean basal expression level (black vertical bars).

Single cell p53 expression varied markedly between samples (figure 3). The four dissociated xenografts measured in the MAC chip that did express p53 derived from two patients, and xenograft material from each of these patients was tested twice: once as a freshly harvested tumour and once as thawed cryopreserved cells (figure 4). All four samples showed a similar distribution of expression, with the majority of cells showing low or undetectable ‘basal level’ of expression, and a few outlier cells expressing comparatively larger quantities of p53. The repeated samples PDX-98-P4 and PDX130-P2 showed similar distributions between samples, though there was heterogeneity in the highly expressing cells. In total outlier cells represented between 1.7% and 5.8% of the total single cells analysed (table 2). Relative levels of p53 expression in outlier cells can be several orders of magnitude higher than the mean level of expression in the basal distribution (figure 5). In the most extreme case, an outlier cell produced a p53 response in the assay some 493  ×  higher than the mean of the basal population. Even within these four expressing PDXs there were high proportions of cells (8, 54, 62 and 94%) in which p53 could not be measured. It is likely that these cells express a low level of p53 which is below the detection limit of the MAC chip assay.

Outlier cells from both freshly harvested xenograft samples expressed more p53 than their frozen counterparts, with the mean expression of the frozen outliers reducing to 20% and 34% of PDX98-P4 and PDX130-P2 respectively. Trypan blue was used as the viability indicator for the earlier fresh experiments and as has already been noted, this can generate a short term increase in p53 expression levels. Alternatively, the freeze-thaw cycle may affect p53 levels and this will require further study. Additional complexity is added to interpretation of the results when one considers the basal cells: in PDX98-P4 the mean basal expression dropped for the frozen material, whereas for PDX130-P2 it increased.

**Figure 6.**
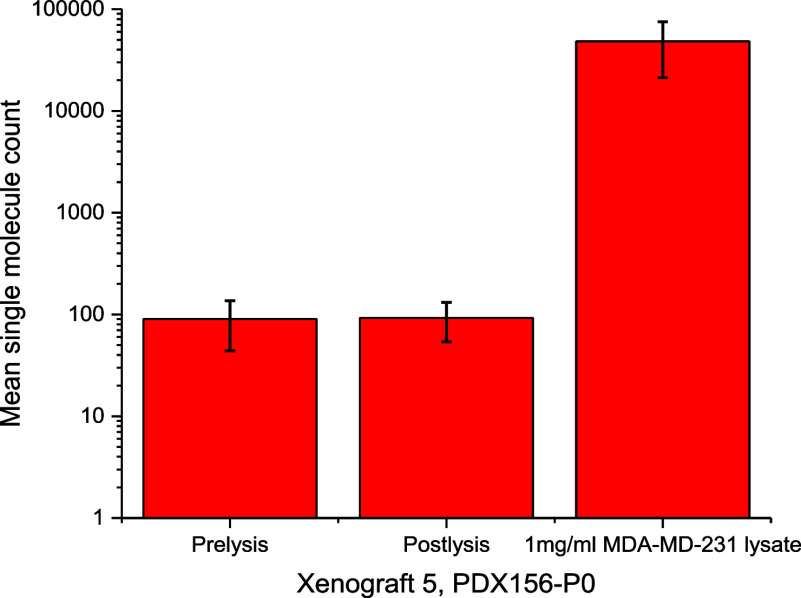
Introduction of positive p53 control into control lanes of the Standard Candle chip confirms the MAC chip assay is operational when no p53 is measured in the sample. p53 positive MDA-MD-231 lysate at 1 mg ml^−1^ total protein (~8 cells nl^−1^) is introduced to the chip via the secondary inlet after the single cell experiment is performed. This is not performed concurrently with the main experiment in order to avoid the possibility of diffusion of the target analyte from the main reservoir into the single cell analysis chambers and artificially increasing the signal measured from the sample cells. Single molecule counts are registered prior to lysis due to non-specific binding of the fluorescent secondary antibody to the primary antibody and surrounding functionalised coverslip.

Five PDX samples derived from four separate patients showed no detectable p53. To confirm the assay was functional in these cases a positive p53 control was added to the chip via the secondary inlet (figure 6). For PDX156-P1 this was likely due to the truncating TP53 mutation at R342 which prevents formation of the full length protein. This truncation leads to the loss of one of the antibody binding epitopes required for the sandwich assay. The p53 assay is an antibody sandwich system which requires the anti-p53 antibodies’ respective N- and C-terminal target epitopes to be available to bind in order for a signal to be generated at the surface of the coverslip. As the necessary C-terminal epitope is not present in p53 with a R342 truncation, the assay will fail, and no protein can be measured even if the variant is highly expressed. We have observed similar null signals for the p53 MAC chip assay with p53-knockout cells of the HCT116 colorectal carcinoma cell line (data not shown). However, the lack of expression in the remaining three cases could not be explained by p53 mutations alone. For the only PDX which was analysed on two different occasions (PDX97-P2; each time as freshly harvested tumour material) no p53 signal was measured either time the assay was performed.

It is interesting to note that PDX73-P2 harboured the same R175H p53 mutation as PDX130-P2 and yet had unmeasurable levels of p53; a confirmation that protein expression assays can reveal information which cannot be assessed by sequencing the gene. R175H and other missense mutations typically occur in the DNA binding domain of p53 and therefore prevent p53 from binding to its target genes. However, it has been shown that specific missense mutations, including R175H, have additional gain of function effects that can induce resistance to chemotherapy agents and result in highly metastatic cancer [29]. R175H mutants usually overexpress p53, though the mutant tetramers are inhibited in their binding to the target genes [30]. This documented overexpression in R175H mutants is a tempting explanation for the comparatively high basal level of PDX130 samples, however the unmeasurable levels of p53 in PDX73 leave this an open question at this stage. Mutant p53 tetramers can still interact with other proteins via the transactivation domains, and it has been shown that these interactions may inhibit other tumour suppressor proteins, leading to increased resistance to chemotherapy [31, 32].

**Table 2.** Numerical summary of p53 as quantified by the MAC chip assay in PDX samples. Clearly the presence of a large number of samples below the sensitivity of the assay skews the basal mean, and is meant purely to illustrate the extreme nature of the outlier cells.

	Basal cells			Outlier cells			% outlier	% null
	Number of cells	Mean	SD	Number of cells	Mean	SD		
PDX98-P4	71	24	63	2	8908	3939	2.7	61.6
PDX98-P4^*^	49	0	0	3	1790	474	5.8	94.2
PDX130-P2	132	36	92	4	4553	1277	2.9	53.7
PDX130-P2^*^	59	185	145	1	1527	—	1.7	8.3

### 3.2. phospho-p53 (S15) measurements

**Figure 7.**
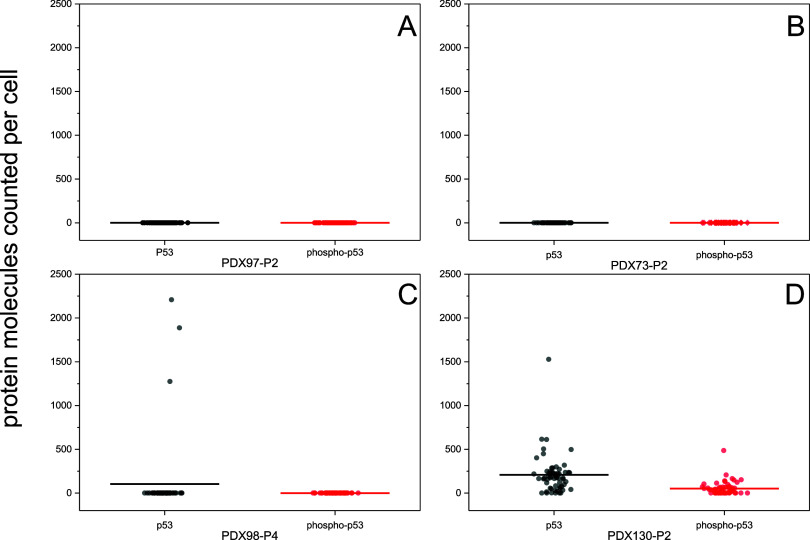
p53 and phospho-p53 responses of the PDX samples which underwent multiplexed MAC chip analysis. The emergence of categorical response types may be visible in multiplexed data, p53/phospho-p53 (−/−) as in (A) and (B); (+/−) as in (C); and (+/+) as in (D).

**Figure 8.**
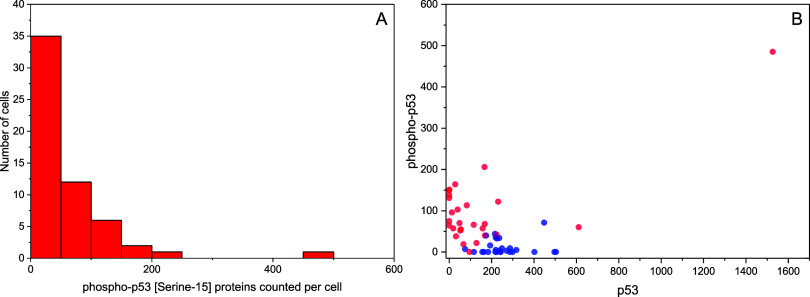
(A) Phosphorylated p53 distribution of PDX130-P2^*^ and (B) relationship between phosho-p53 and p53 in single cells. Each data point represents a single cell from PDX130-P2^*^ lysed in the MAC chip. Red data points  =  MAC chip 1, blue data  =  MAC chip 2, cells lysed approximately 4–5 h after MAC chip 1.

Multiplexed measurements were conducted on four PDX samples derived from four distinct trial patients: three from archived frozen stocks (PDX73-P2, PDX98-P4^*^ and PDX130-P2^*^), and the other a freshly harvested tumour (PDX97-P2^**^). Similar to total p53 quantification, samples varied in the quantity of phosphorylated p53 molecules that were detected (figure 7). Firm conclusions cannot be made due to the small number of samples tested, but we note with caution that it may already be possible to identify three distinct patterns within the analyte distributions—that of p53/phospho-p53 (+/+); (+/−), and (−/−). Interestingly phospho-p53 levels in PDX130-P2^*^ display a long-tailed distribution as observed for p53. Figure 8(B) shows the trend of phospho-p53(S15) versus p53 in single cells from PDX130-P2^*^. The lack of overlap in phosphorylation levels between the two MAC chips may be a consequence of the significant time delay between lysis in the two assays.

Note that there is no inconsistency in measuring phospho-p53 in the assay when no p53 is registered as the signal is not calibrated for absolute quantification; what it reflects is that the anti-pS15-p53 secondary antibody is of higher affinity than anti-p53 secondary, and also has a lower rate of non-specific binding to the printed primary p53 antibody.

## 4. Discussion

We have demonstrated that single cell-resolution proteomic information can be extracted with the MAC chip technology from PDX material. The heterogeneity we have observed within and across samples is notable, though the data itself is early stage, and it is not yet possible to develop a firm biological narrative. A more comprehensive investigation of this is required in order to fully describe the phenomenon and assess its implications. Demonstrating these protocols and workflow on xenograft tissue suggests that our method can also be directly applied for single cell analysis of primary tumour material, without a need to expand the tissue in a host organism.

Basal expression in several PDX samples was below the limit of what was quantifiable by the assay. This was surprising as for the p53 and phospho-p53 assay a 4.5 nL chamber is sufficient to allow the reliable detection and quantification of p53 in both BE (highly expressing) and MCF-7 (low-expressing) cell lines [15]. At ~1–2  ×  10^2^ molecules the limits of detection in these experiments were in line with previously published values for the p53 assay, and were constrained by the variation in non-specific binding of the secondary antibody [14, 17]. For low-abundance proteins the dimensions of the chamber may be reduced so as to concentrate the analyte and increase the favourability of binding at the antibody capture spot [15]. Migrating to a MAC chip with smaller chambers may therefore confer a gain in assay sensitivity that allows discrimination of the p53 expression in the low level basal populations.

The limiting step in the current protocol for microscopic sampling of a solid tumour is the dissociation procedure, which according to the manufacturer is recommended for tumours down to 0.01 g (~10 mm^3^). The actual tumour volume processed on a single MAC chip is around 200  ×  smaller than this, and so in the future therefore it may be desirable to develop a microfluidic tumour cell dissociator capable of working in series with the MAC chip and efficiently handling and dissociating tumour volumes less than those capable by the current state of the art in commercial kits. We note that recently others have already begun to prototype such devices [33].

A decrease in the expression level of p53 in the outlier cells in the cryopreserved samples was noted above. This may be due to inherent differences between fresh and cryopreserved material, but it should be noted that other physical factors could account for the discrepancy in the measurements, for instance variations in the mean antibody spot area between chips, which may vary depending on microarray printing conditions such as relative humidity, pin head fouling, surface hydrophobicity and contact angle, as well as MAC-chip specific issues with the printed spot such as damage or partial destruction during alignment of the microarray slide with the PDMS channels of the microfluidic. These factors would not account for the variation in the basal cells, which decreased for one sample and increased for the other. If cryopreservation does lead to analyte copy number variations, then a standard protocol would address this in clinical usage, e.g. using only fresh biopsy material for analysis. Nevertheless, we have demonstrated that single cell protein analysis can be successfully performed on samples that have been cryopreserved.

It has been proposed previously that p53 expression can be used as a biomarker for prognostic outcomes in cancer patients, though studies are conflicting on its usefulness [34]. Although overexpression is commonly observed in 60–80% of colorectal carcinoma, few studies have shown any statistical significance between p53 overexpression and survival outcomes [35]. Importantly, bulk quantification assays applied to cell lines or cancer samples lack the ability to discriminate whether protein expression is altered across all cells or whether this is driven by heterogeneous changes affecting only a fraction of cells in a sample. Single cell analysis in a MAC chip confers several advantages that may get around these shortcomings. Firstly, the sub-populations within the cell population can be selected using optical trapping based upon cell surface biomarkers, such as EpCAM, as demonstrated in this work. Secondly, an activated form of p53, pS15-p53 can be detected and its ratio to total p53 levels quantified. Thirdly, single molecule-sensitive readouts may provide the sensitivity required to differentiate between clinically relevant p53 responses. Finally, the MAC chip can work with only a few hundreds of cells, and so can be applied to rare samples in a way that more materially demanding methods cannot.

By detecting single cell pS15-p53 we have a detected an activated subset of p53 that is key to its cellular function, and as such the MAC chip can be considered an activity assay. The p53 activation pathway is exceptionally complex, and fully accounting for the expression levels observed in the samples analysed here is impossible given the limited size of the cohort. However, we note that an individual cell that expresses high levels of p53 without any S15 phosphorylation, may have a completely different phenotypic effect to a cell that expresses low levels of p53 but which is highly phosphorylated, and therefore highly active [27, 31, 36–43]. Additionally, phosphorylation in mutated isoforms can have different functional implications to that of activated wild-type protein, and there is already evidence to support the idea of differential gain of function with respect to mutant p53 phosphorylation [44]. This may be something the multiplexed MAC chip can directly measure in terms of relative or absolute copy number ratios, and as such the technology may allow elucidation of the causes of phenotypic effects seen in different cancer samples. Implementing this methodology into a clinical trial of greater scope has the potential to identify clinically meaningful patient subtypes with respect to p53 where other methods have not.

We have demonstrated the work here on PDX samples, but the protocol can be applied to disaggregated clinical tumour material of almost any source, and is particularly suited to scarce samples limited by the quantity of cells available. The small demand for primary material and the ability to work with extremely low viability samples means that the MAC chip is an obvious candidate assay for the proteomic profiling of cytology samples acquired through fine needle aspirates (FNAs). The low number of cells required also means that for larger sources of clinical material, the MAC profiling could easily be done in parallel to other types of assays without placing a significant restriction on the quantity of material available. A MAC chip assay can in principle be developed for any protein of interest provided there are antibodies (or other capture agents such as DNA or small molecule covalent binders) of sufficient affinity available for that target. Because p53 is not a routine biomarker in therapeutic oncology, the results here should be viewed as a proof-of-principle which shows the potential of the technique for translational single cell proteomics. Development of other assays would allow quantification of proteins with immediate clinical relevance, such as steroid hormone or growth factor receptors. For example, a HER2 assay could examine expression level heterogeneity in HER2 positive breast or gastric cancer and could be assessed for correlation with treatment outcomes for trastuzumab, an anti-HER2 antibody drug, which is routinely applied to these patients. Furthermore, receptor expression heterogeneity in circulating tumour cells (CTCs) has been identified as a possible mechanism of acquired resistance to treatments targeting estrogen receptor and epidermal growth factor receptor (EGFR) in breast and colorectal cancer respectively [45,46]. Characterising this heterogeneity with high resolution is important for the identification of clear subpopulations, and this is something MAC chips can address in CTCs as well as the tumour itself. Others have already envisaged individualised therapy programs informed by single cell proteomics and test panels of anti-cancer agents [10]. *Ex vivo* screens of routinely used and experimental therapeutic compounds are possible in the MAC chip through an extension of the described protocol that incorporates cell drugging within the microfluidic chip prior to analysis. Potentially this could be a vital approach to guide drug selection for personalized cancer therapy as it would allow patient material to be assessed for heterogeneous single cell responses with respect to the drug and enable targeted therapeutic interventions which take into account the inherent heterogeneity of the tumour.

While producing assays of sufficient sensitivity for multiple analytes is challenging, multiplexed analyses as demonstrated here may allow the patient stratification space to be defined with even greater clarity. It may well emerge that it is the relative degree of these responses that is critical for the development of strategies for therapeutic intervention, and it is this that the high-resolution proteomic data that MAC chip analysis provides is uniquely situated to address.

To conclude, we have demonstrated that the MAC chip workflow is a practical method for the acquisition of single molecule-resolution single cell data in very small quantities of clinical tumour samples. Expanding the repertoire of analytes for which there are MAC chip assays beyond p53 and its phosphorylated form must now be a priority if the technique is to translate to the oncology clinic. Longitudinal studies are now required where tissue heterogeneity is monitored with the MAC chip through the course of treatment such that the clinical utility of the platform can be assessed. The uniquely quantitative single cell information provided in such studies may reveal insights unobtainable by other methods.
